# NGF-releasing Prussian blue nanoparticles for nerve injury repair of lumbar disc herniation

**DOI:** 10.3389/fchem.2024.1503330

**Published:** 2024-12-18

**Authors:** Xiaoxing Tang, Xin Sun, Yun Ji, Xuehua Huang, Shilin Xiao, Yanjing Zhou, Ke Ma, Hongjie Yuan

**Affiliations:** ^1^ Department of Radiology, Nantong Hospital of Traditional Chinese Medicine, Nantong Hospital to Nanjing University of Chinese Medicine, Nantong, China; ^2^ Department of Anesthesiology, Affiliated Hospital 2 of Nantong University, Nantong, China; ^3^ Department of Algology, Xinhua Hospital, Shanghai Jiaotong University School of Medicine, Shanghai, China

**Keywords:** lumbar disc herniation, Prussian blue nanoparticles, nerve growth factor, neuron-like PC12 cells, nerve injury repair

## Abstract

**Introduction:**

Compression of the nerve root by a lumbar disc herniation can cause radiating pain in the lower limbs, and the nerve root decompression treatment may leave some patients with motor dysfunction and reduced sensory function. Studies have shown that nerve growth factor (NGF) can promote nerve growth and repair, but high doses, long duration, and immune response have become bottlenecks of its clinical application.

**Methods:**

To overcome this obstacle, we developed Prussian blue (PBs) nanoparticles with the bio-delivery function and antioxidant effects of nanoenzymes. NGF was conjugated to the surface of PBs nanoparticles (PBs-NGF), which can be directly delivered to nerve cells.

**Results:**

The results showed that free PBs showed great advantages in scavenging oxygen free radicals and antioxidants, while PBs-NGF showed good biocompatibility. At the cellular level, cell proliferation assay and fluorescence microscopy analysis confirmed that PBs-NGF significantly promoted the proliferation, differentiation, and neurite outgrowth of neuron-like PC12 cells compared with free NGF. In a nerve root compression (NRC) rat model, behavioral observations (paw withdrawal threshold, PWT, and paw withdrawal latency, PWL) confirmed that PBs-NGF eased the pain caused by nerve root compression. H&E staining showed that PBs-NGF could significantly reduce the inflammatory infiltration of nerve roots, and ELISA results showed that the concentrations of inflammatory markers (IL-6, IL-1β, and TNF-α) were also significantly reduced.

**Conclusion:**

In summary, the developed functional nanoplatform provides a basis for the clinical application of NGF in lumbar nerve root injury with disc herniation compression and a new treatment strategy for patients.

## 1 Introduction

Lumbar disc herniation compresses the lumbar nerve root, a major cause of nerve injury, sensory dysfunction, and motor dysfunction in lower limbs (Hasvik, Haugen, and Grøvle, 2021). The peripheral nerve fibers have self-regenerative potential. For most patients, bed rest, traction, and surgery to relieve the pressure can allow good recovery of nerve function (Goto and Inoue, 2023; Hoz et al., 2021). However, for many patients with severe nerve compression, long compression time, or underlying diseases such as diabetes, the recovery of nerve function is often not satisfactory (Rogerson, Aidlen, and Jenis, 2019). Therefore, it is of high clinical significance to explore the mechanism of poor recovery of nerve root function in lumbar disc herniation and to develop targeted therapeutic drugs.

Nerve growth factor (NGF) is an important regulator of neuronal differentiation, growth, survival, and death (Gu et al., 2020). Studies have shown that reduced NGF transport produced by NGF target tissue/innervated tissue can lead to nerve cell damage (Aloe et al., 2015). In experimental animals and on isolated cells, NGF protected not only the survival of degenerates’ peripheral nerve cells but also the regulation of neurotransmitter and neuropeptide synthesis in sympathetic and sensory nerve cells (Wang et al., 2022a; Wang et al., 2022b; Wang et al. 2020). The use of exogenous NGF affected neuroplasticity, enabling the adult nervous system to change its structure and function in response to stimulation (Sims et al., 2022). Exogenous NGF can promote the growth of peripheral nerves and restore the functional activity of peripheral nerve fibers and damaged neurons (Barker et al., 2020). Therefore, *in vitro* administration of NGF to promote nerve repair may be an effective method for the treatment of nerve root injury caused by intervertebral disc herniation.

At present, high doses, long duration, and induced immune response are the bottlenecks of NGF in promoting nerve repair (Reis et al., 2023; Zhang et al., 2017). In order to solve this problem, researchers hope to develop and apply an NGF delivery system. Nerve root compression increases oxidative stress, which may lead to axonal degeneration and myelin degeneration (Silwal et al., 2023). Reactive oxygen species (ROS) enhance the synthesis of inflammatory cytokines and other inflammatory mediators, which play an important role in inflammatory mechanisms and loss of axonal conductivity (Wang et al., 2018; Jamil et al., 2020; Luo et al., 2021). In addition, ROS also play a crucial role in the initiation and maintenance of regenerative responses in the body (Helston and Amaya, 2021). In addition to ROS, reactive nitrogen oxides, including NO and ONOO-, are involved in the occurrence and development of nerve injury (Ashki, Hayes, and Bao, 2008). According to the mechanism by which oxidative stress is involved in nerve root injury induced by disc herniation compression, nano-enzymes with reactive oxygen and nitrogen species (RONS) enzymatic activity may be the best material to deliver NGF and play a synergistic therapeutic effect.

Prussian blue (PBs) nanoparticles stand out among many nanozymes due to their biological safety (Li et al., 2022). PBs have been widely used as disease therapeutic agents and biosensors due to their excellent magnetic properties, photothermal conversion, and multi-enzyme mimetic capabilities (Song et al., 2021; Lu et al., 2023; Komkova and Karyakin, 2022). Studies have reported that PBs can effectively scavenge ROS, including OH, H_2_O_2_, and OOH, through the activities of peroxidase (POD), catalase (CAT), and superoxide dismutase (SOD) (Gao et al., 2020; He et al., 2023; Estelrich and Busquets, 2021). Therefore, PBs may have great potential in treating diseases that target ROS (Zhao et al., 2018). In the study of ischemic stroke disease, PBs have shown a neuroprotective effect by clearing RONS (Zhang et al., 2019). However, to date, PBs-based nanomedicines have not been explored and applied in the treatment of nerve root injury caused by lumbar disc herniation.

**FIGURE 1:**
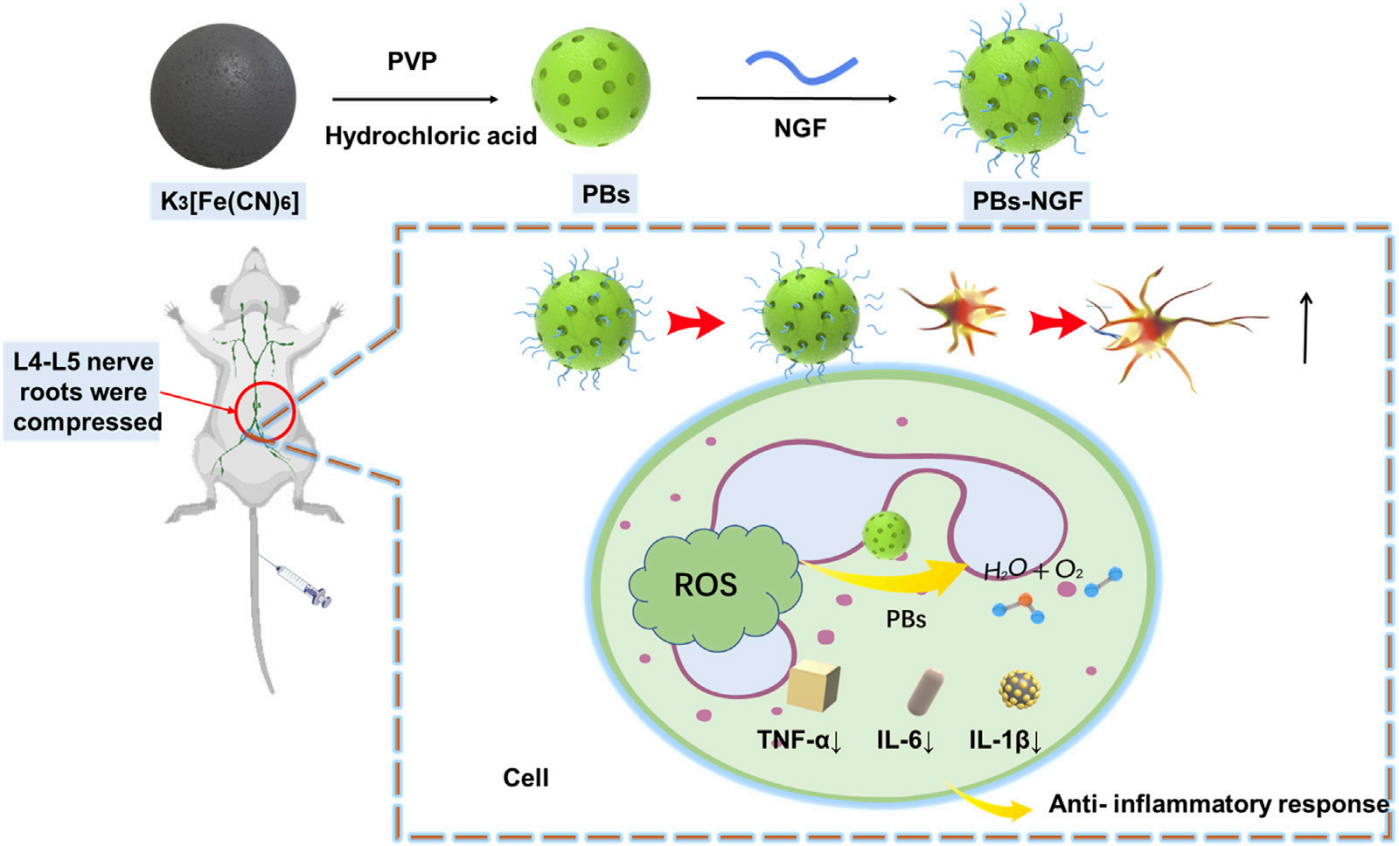
Schematic diagram of the preparation of PBs-NGF.

In the present study, NGF was uniformly coupled to the surface of PBs for nerve injury repair (Figure 1). The data showed that the PBs-NGF had significant dimensional stability and biocompatibility and also possessed an excellent sustained release function for NGF. PBs-NGF can deliver pain relief and repair the effect of nerve damage with lower systemic toxicity. They are expected to be an effective treatment for nerve injury repair.

## 2 Materials and methods

### 2.1 Materials

Poly (vinylpyrrolidone) (PVP, K30), hydrochloric acid (HCI, 36%), potassium ferricyanide (K3 [Fe(CN)6]) 3,5,3′,5′-tetramethylbenzidine (TMB), hydrogen peroxide (H_2_O_2_, 30%), HAc-NaAc, and TiO2 were obtained from Sigma-Aldrich (United States). Nerve growth factor (NGF) was obtained from Cell Guidance Systems (United States). 5-tert-butoxycarbonyl-5-methyl-1-pyrroline-N-oxide (BMPO) was purchased from MERYER (China).

### 2.2 Synthesis of Prussian blue (PBs) nanoparticles

PVP (10.0 g) and K3 [Fe(CN)6] (870 mg) were dissolved into a hydrochloric acid solution (1 M, 50 mL) under magnetic stirring for 60 min. The mixture was heated at 100°C for 24 h. Finally, the solution was centrifuged to obtain Prussian blue nanoparticles.

### 2.3 Characterization

The morphology of PBs was observed by transmission electron microscopy (TEM, JEM-2100, Japan). Scanning electron microscopy (SEM) images were obtained using an Ultra Plus microscope (Carl Zeiss, Germany). The size and surface charge of PBs were analyzed using dynamic light scattering (DLS, NanoZS90, Malvern, England).

PBs were dispersed in DI water and RPMI-1640 medium for 12 h, 24 h, and 72 h. The size was analyzed using dynamic light scattering.

### 2.4 Loading and release of NGF

PBs (5 mg) and NGF (2 mg) were dissolved in 100 mL phosphate buffer (PBS) solution and stirred for 6 h at room temperature. The PBs-NGF were obtained after centrifugation. Loading efficiency (%) = the amount of NGF encapsulated/(weight of PBs-NGF) × 100.

The release of NGF was studied using dialysis. Briefly, the NGF solution and PBs-NGF nanoparticle solution were put into a dialysis bag. The dialysis bag was placed in 0.01 M PBS (40 mL, pH 7.4) with 5% BSA, which was stirred at 100 rpm and 37°C. At predetermined intervals, 1.0 mL of the release buffer was collected, and 1.0 mL of fresh PBS was added. The concentration of NGF in the solution was detected, and the release rate of NGF was calculated.

### 2.5 Detection of peroxidase-like activity

PBs (6 μg) and TMB (10 mg) were dispersed in 0.2 M HAc-NaAc (22 mL). Then, 3 mL of 30% H_2_O_2_ was added to the mixture. The absorbance at 650 nm was detected at a certain reaction time via a multifunctional microplate reader (Varioskan ALF, Thermo Scientific, United States).

### 2.6 Detection of the effect of PBs on H_2_O_2_

Phosphate buffer (pH 7.4) was supplemented with 2 mL of 30% H_2_O_2_ (1.2 M), followed by 40 μg of PBs. Oxygen generation was analyzed with the oxygen electrode of the multifunction analyzer (BDO-200A, BELL, China).

### 2.7 Detection of the effect of PBs on hydroxyl radicals

Various concentrations of PBs were added to 50 mM BMPO containing 0.1 mg/mL TiO_2_. The mixed solution was added to a quartz capillary and exposed to UV light at 340 nm for 5 min. Electron spin resonance (ESR) spectra were detected via a Bruker EMX spectrometer.

### 2.8 Detection of the effect of PBs on superoxide anions

Different concentrations of PBs were added to the xanthine/xanthine oxidase system. Then, 50 mM BMPO was added to the mixture. Finally, a Bruker EMX spectrometer was used to obtain electron spin resonance (ESR) spectra.

### 2.9 Cell culture

PC12 cells were purchased from ATCC and cultured in RPMI-1640 medium supplemented with 10% fetal bovine serum (FBS) and 1% penicillin–streptomycin (P/S) at 37°C, 5% CO_2_.

### 2.10 CCK-8 assay

PC12 cells (1 × 10^4^) were inoculated on 96-well plates in an incubator at 37°C and 5% CO_2_. The cells were treated with different concentrations of PBs (0 μg/mL, 12.5 μg/mL, 25 μg/mL, 50 μg/mL, 100 μg/mL, 200 μg/mL, and 400 μg/mL) for 24 h. Then, 10 μL CCK8 reagent (MCE, United States) was added to incubate for 2 h. Finally, the absorbance value was detected at 450 nm via a multifunctional microplate reader.

### 2.11 Hemolysis assay

Several 200-μL aliquots of different concentrations of PBs and PBs-NGF (0 μg/mL, 12.5 μg/mL, 25 μg/mL, 50 μg/mL, 100 μg/mL, 200 μg/mL, and 400 μg/mL) were dispersed in mouse blood (1.3 mL). The mixture was incubated in a water bath at 37°C for 1 h. After centrifugation, 200 µL of the supernatant was added to a 96-well plate, and the absorbance was measured at 540 nm using a microplate reader.

### 2.12 Western blot

Total protein was extracted from PC12 cells with RIPA buffer (Thermo Fisher Scientific, United States), and protein concentration was determined by a BCA assay (Pierce, United States). A 20-μg protein sample was separated by 10% SDS-PAGE and transferred to PVDF membranes (Millipore; Burlington, MA, United States). Subsequently, the membranes were blocked with 5% BSA (Sigma-Aldrich, United States) and then incubated with anti-NeuN antibody (1:1000, ab177487, Abcam, United States), anti-DCX antibody (1:1000, ab18723, Abcam, United States), and anti-actin antibody (1:1000, ab8227, Abcam, United States) overnight at 4°C. After incubation, the membranes were washed three times with phosphate buffer (PBST) and incubated with horseradish peroxidase (HRP)-conjugated secondary antibody (1:1000, ab97051, Abcam, United States) for 2 h at room temperature. The membranes were then washed three times with PBST and exposed using enhanced chemiluminescence detection (Pierce, United States).

### 2.13 Induced differentiation of PC12 cells

PC12 cells were inoculated in Type I collagen-coated culture plates at 37°C and 5% CO_2_ incubator overnight. The cells were divided into four groups: a normal group, an NGF group, a PBs group, and a PBs-NGF nanoparticle group. NGF, PBs, or NGFs-PBS were added to the culture medium to incubate the cells for 4 days, respectively. The differentiation of PC12 cells was observed under a microscope (Leica DMi1, Germany).

### 2.14 Nerve root compression (NRC) model

Healthy Sprague–Dawley rats, 6–8 weeks old, 200–250 g, were purchased from SPF Biotechnology Co., Ltd. Nerve root compression surgery was performed in accordance with a previous study (Obata et al., 2002). The Sprague–Dawley rats were anesthetized by intraperitoneal injection of pentobarbital sodium (40 mg/kg). The rat’s back hair was shaved, and the tail was cut using sterile surgical instruments to obtain nucleus pulposus (2 mg) and annulus fibrosus (2 mg) from the tail disc. In the model group, the left L4 and L5 nerve roots were exposed after hemilaminectomy, and the autologous intervertebral disc tissue was implanted into the exposed nerve roots (L4, 5). Then, the wound was rinsed with saline and closed in layers with 3–0 silk sutures, allowing the muscles at the top to immobilize the disc tissue. Rats in the sham-operated group had autologous discs removed after hemilaminectomy, but no autologous disc tissue was implanted. All animal experiments were conducted with the approval of the Nantong Hospital Traditional Chinese Medicine Ethics Committee.

### 2.15 Effect of PBs on an NRC rat model

Sprague–Dawley rats were divided into five groups: sham group, NRC group, NRC + NGF group, NRC + PBs group, and NRC + PB-NGF group. Rats in the NGF, PBs, and PBs-NGF groups were injected intravenously twice a day from days 2–4 subsequent to NRC surgery. Paw-withdrawal threshold (PWT) and paw-withdrawal latency (PWL) behaviors were detected. After 21 days of treatment, the rats were euthanized, and the nerve roots were removed for H&E staining.

### 2.16 Behavioral tests

PWTL was measured by applying a von Frey filament to the plantar surface of each hind paw (Tal and Bennett, 1994). In brief, after 20 min of acclimatization, von Frey filament (0.41 g, 0.70 g, 1.20 g, 2.04 g, 3.63 g, 5.50 g, 8.51 g, and 15.14 g) stimulation was performed on the plantar surface of the hind paw of rats. At the end of each round of stimulation, the paw withdrawal threshold was taken as the force required to trigger the hind paw reflex. The total number of paw withdrawal events was calculated for each rat.

The testing procedure for PWL was performed according to a previously published method (Hargreaves et al., 1988). Briefly, after 15 min of acclimatization, the plantar surface of the hind paw was stimulated by continuous infrared heat. The time to trigger the paw withdrawal reflex was recorded. A cutoff time of 25 s was set to prevent damage to the paws.

### 2.17 Enzyme-linked immunosorbent assay (ELISA)

TNF-α, IL-1β, and IL-6 levels in the nerve roots were measured by ELISA. The tissues were homogenized in the PBS solution. The homogenized samples were subsequently centrifuged at 10,000 *g* for 30 min at 4°C. Supernatants were assayed using the manufacturer’s instructions for the rat TNF-α, IL-1β, IL-6, and ELISA kits (Shanghai Enzyme-linked Biotechnology Co., Ltd., China).

### 2.18 Biological safety assay

Healthy male Sprague–Dawley rats aged 6–8 weeks were injected intravenously with PBs and PBs-NGF. Blood was then taken from the ocular vein for routine blood tests and biochemical analysis. The heart, liver, spleen, lung, and kidney were extracted for H&E staining.

### 2.19 Statistical analysis

Quantitative data are presented as the mean ± SD. Data analyses were performed using GraphPad Prism statistical software. The differences between the two groups were compared using one-way ANOVA or Student's t-test. The differences were deemed statistically significant at *p* < 0.05.

## 3 Results and discussion

### 3.1 Characterization of PBs and release of NGF *in vitro*

**FIGURE 2:**
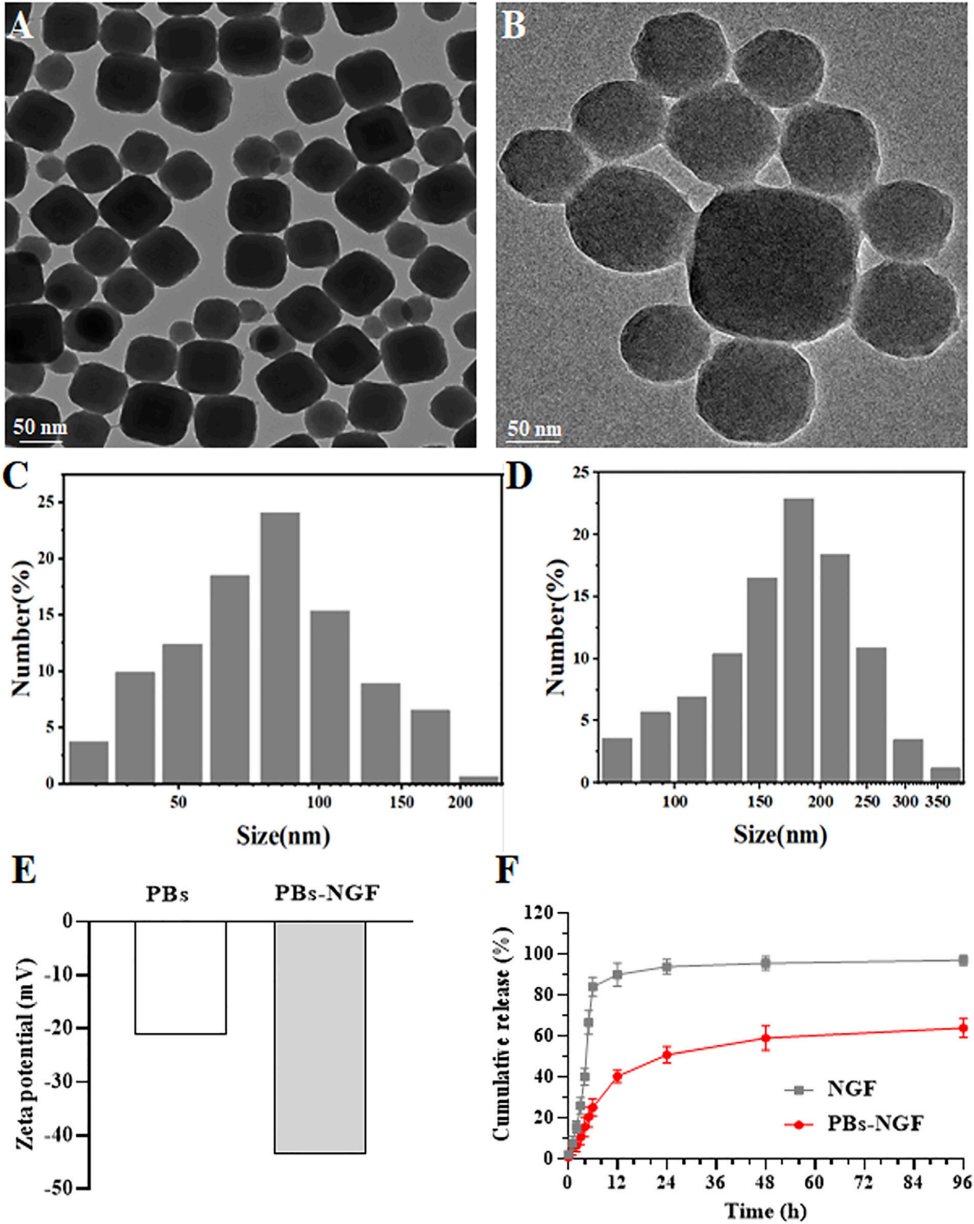
TEM images of PBs **(A)**. SEM images of PBs **(B)**. DLS results of PBs **(C)** and PBs-NGF **(D)**. Zeta potential data of PBs and PBs-NGF **(E)**. NGF release percentage of free NGF or PBs-NGF in PBS solution with 5% BSA **(F)**.

Based on previous literature, polyvinylpyrrolidone (PVP)-modified Prussian blue nanoparticles (PBs) were prepared using a simple hydrothermal method (Zhao et al., 2018). Transmission electron microscopy (TEM) results revealed that the prepared PBs were square and spherical, and the average particle size was ∼80 nm (Figure 2A). Scanning electron microscopy (SEM) images showed that uniform PBs were formed with good monodispersion (Figure 2B). Dynamic light scattering (DLS) was then used to detect PBs, and the particle size changed after NGF coupling on its surface (PBs-NGF). Figures 2C,D showed that the particle size of PBs was consistent with that of TEM, which was ∼80 nm, while the particle size increased to ∼180 nm after surface coupling with NGF. In addition, owing to the negative charges of NGF, the zeta potential of the PBs decreased further after coupling the NGF (Figure 2E). This is similar to the result of zeta potential change after loading NGF with mesoporous silica nanoparticles (Sun et al., 2016). The results primarily indicated the successful construction of PBs-NGF.

*In vitro* release of NGF in PBS with 5% BSA to mimic physiological conditions in blood was further investigated using the dialysis method by increasing the size of the dialysis unit (molecular weight cutoff value of 300 kDa) and adding the release media PBS and BSA. NGF can freely through dialysis membrane. The results are shown in Figure 2F. The recovery rate of close to 100% of free model NGF and PBs-NGF showed the characteristics of slow release. The PBs-NGF released ∼60% of the NGF within 96 h.

### 3.2 Multiple enzyme-like activities of PBs and biocompatibility of PBs-NGF

**FIGURE 3:**
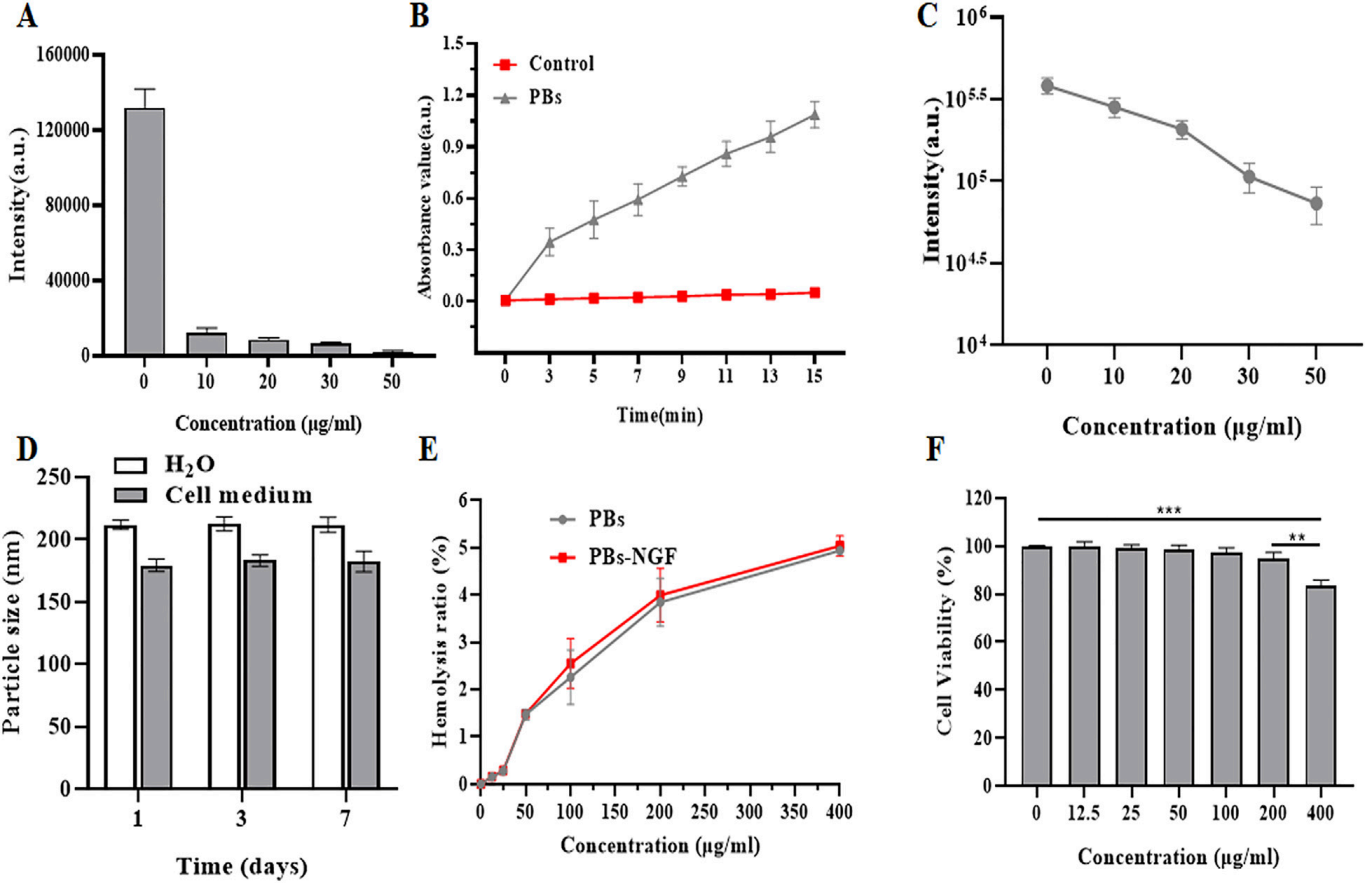
Effect of PBs on ·OH generated by a TiO_2_/UV system. The signal intensities of the second line in the BMPO/·OH spectrum are disclosed **(A)**. POD-like activity of PBs **(B)**. Effect of PBs on ·OOH produced by the xanthine/xanthine oxidase system. The signal intensities of BMPO/·OH are shown **(C)**. Size variations of PBs-NGF dispersed in H_2_O and cell medium **(D)**. Hemolysis rate of PBs-NGF **(E)**. Cell viability of PC12 cells after treatment with PBs-NGF at different concentrations **(F)**. **p < 0.01; ***p < 0.001. N = 3 of each group.

In recent years, PBs have been found to have catalase, superoxide dismutase, and peroxidase-like activities and are highly effective RONS scavengers (Zhang et al., 2021). PBs’ reactive oxygen scavenging ability was determined using the generated hydroxyl free radical system of TiO_2_/UV detection. The results showed that the BMPO/·OH signal strength fell sharply with the increase of concentration of PBs, suggesting that the PBs have a good ability to remove hydroxyl radicals (Figure 3A). Then, the natural peroxidase (POD) substrate 3,5,3,5-tetramethyl benzidine (TMB) was used to detect the POD activity of the PBs. As shown in Figure 3B, the absorbance at 650 nm showed that the absorbance value increased with time after adding PBs, indicating that they had POD-like activity. The results of the xanthine/xanthine oxidase system showed that the signal intensity of superoxide radicals (·OOH) decreased significantly with the increase of PBs concentration (Figure 3C), suggesting that PBs can act as a nano-enzyme to remove ·OOH. The above experimental results indicated that PBs have good antioxidant activity. Zhou et al. also showed that synthetic Prussian blue nanoparticles (PBNPs) could alleviate oxidative stress and increase the activity of intracellular antioxidant enzymes such as superoxide dismutase 1 (SOD1) in intervertebral disc degeneration (IVDD) cells (Zhou et al., 2022).

Understanding nanoparticle size variations in various physiological media plays a vital role in determining the particles’ bio-application fate. We investigated the size stability of our nanoprobes toward different biological buffers by DLS. Results showed that PBs-NGF in H_2_O and cell medium have good stability, and performance over 0–7 days at 50°C showed no significant change in nanoparticle size (Figure 3D), confirming the stable size feature of PBs-NGF. When the concentration of these nanoparticles reached 400 μg/mL, the hemolysis rate was slightly increased to 5%, and there was no significant difference in the hemolysis rate between PBs and PBs-NGF (Figure 3E). Finally, the cell viability of PC12 cells infused with a gradient concentration of PBs was detected using a cell counting kit 8 (CCK8). The safe concentration of PBs is below 200 μg/mL (Figure 3F), while cell viability is inhibited at 400 μg/mL. All the above data confirm that PBs have good biocompatibility. Consistent with our results, the Prussian blue nanoparticles (HPBZs) constructed by Zhang et al. (2019) remain within concentration safety at concentrations up to 320 μg/mL.

### 3.3 PBs-NGF promoted PC12 cell differentiation and neurite growth

**FIGURE 4:**
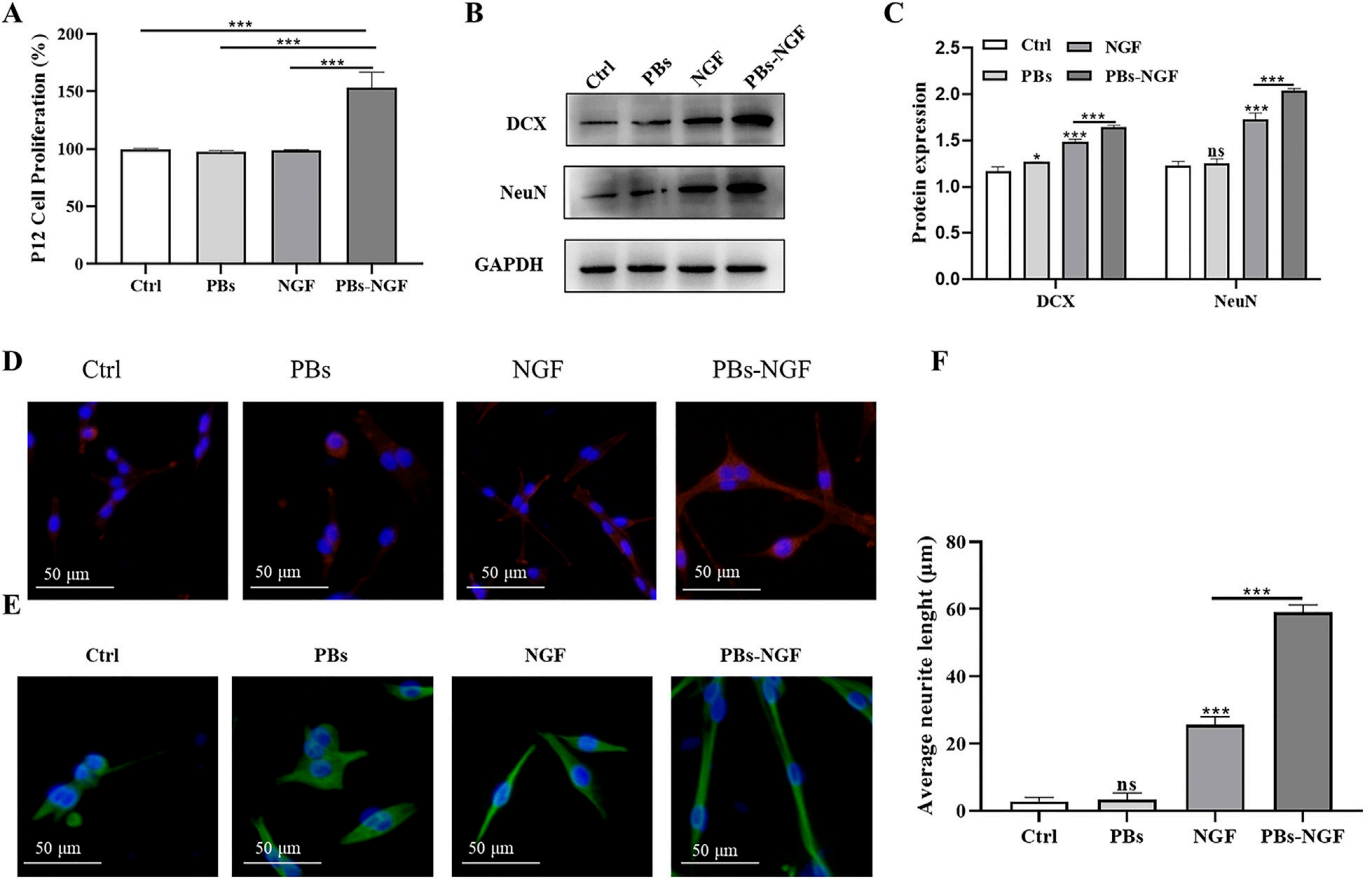
Determination of PC12 cell proliferation after free NGF, PBs nanoparticle, or PBs-NGF nanoparticle treatment with a CCK-8 assay **(A)**. Western blotting analysis of DCX and NeuN in PC12 cells treated with free NGF, PBs, or PBs-NGF **(B)**. Quantification of DCX and NeuN proteins expressed in **(B)**. GAPDH was set as a loading control **(C)**. PC12 cells were exposed to free NGF, PBs, or PBs-NGF (50 μg/mL) for 48 h, and staining with MAP2 (red, **(D)**) and tubulin (green, **(E)**). Microscopic images were acquired using a fluorescence microscope. The growth of neurites was determined using ImageJ software (NIH) **(F)**. *p < 0.05; ***p < 0.001. N = 3 of each group. ns indicates no statistical difference.

The pheochromocytoma cell line (PC12) is a model of nerve cells derived from the adrenal glands. The cells’ uniformity and repeatability allow them to be widely used as a model for the differentiation of neurons (Sultan et al., 2021). PC12 cells depend on NGF and display a typical neuronal phenotype and neurite outgrowth in response to this neurotrophic factor through neuronal cell differentiation (Chua and Lim, 2021). To explore the effect of PBs-NGF on neuronal differentiation, PC12 cells were exposed to soluble free NGF, free PBs, PBs-NGF, or PBs-NGF. To clarify the effect of NGF loading on promoting the differentiation of PC12 cells, the CCK-8 assay was used to detect cell viability after 48 h of exposure. As shown in Figure 4A, PBs loaded with NGF significantly induced higher differentiation of PC12 cells. Then, a Western blot assay was used to detect the protein expression levels of neuron-specific markers doublecortin (DCX) and NeuN to analyze the impact of PBs-NGF on PC12 cell differentiation. Figures 4B,C revealed that, compared with free NGF, PBs-NGF nanoparticle induction significantly increased the protein expression levels of DCX and NeuN in PC12 cells, suggesting that the synergistic effect of PBs and NGF has a better differentiation induction effect. Morphological changes of PC12 cells and the difference in neurite length with PBs-NGF treated for 48 h were observed using MAP-2 and NeuN immunocytochemistry. Figures 4D–F show that there was no neurite extension in the control group, while free NGF and free PBs promote the differentiation and growth of neurite of PC12 cells, with the most significant effect with PBs-NGF. This may be due to the PBs nanoparticle packet loading reducing the rate of NGF degradation, prolonging its half-life, and increasing its availability.

### 3.4 PBs-NGF alleviated pain and improved nerve injury repair in NRC rats

**FIGURE 5:**
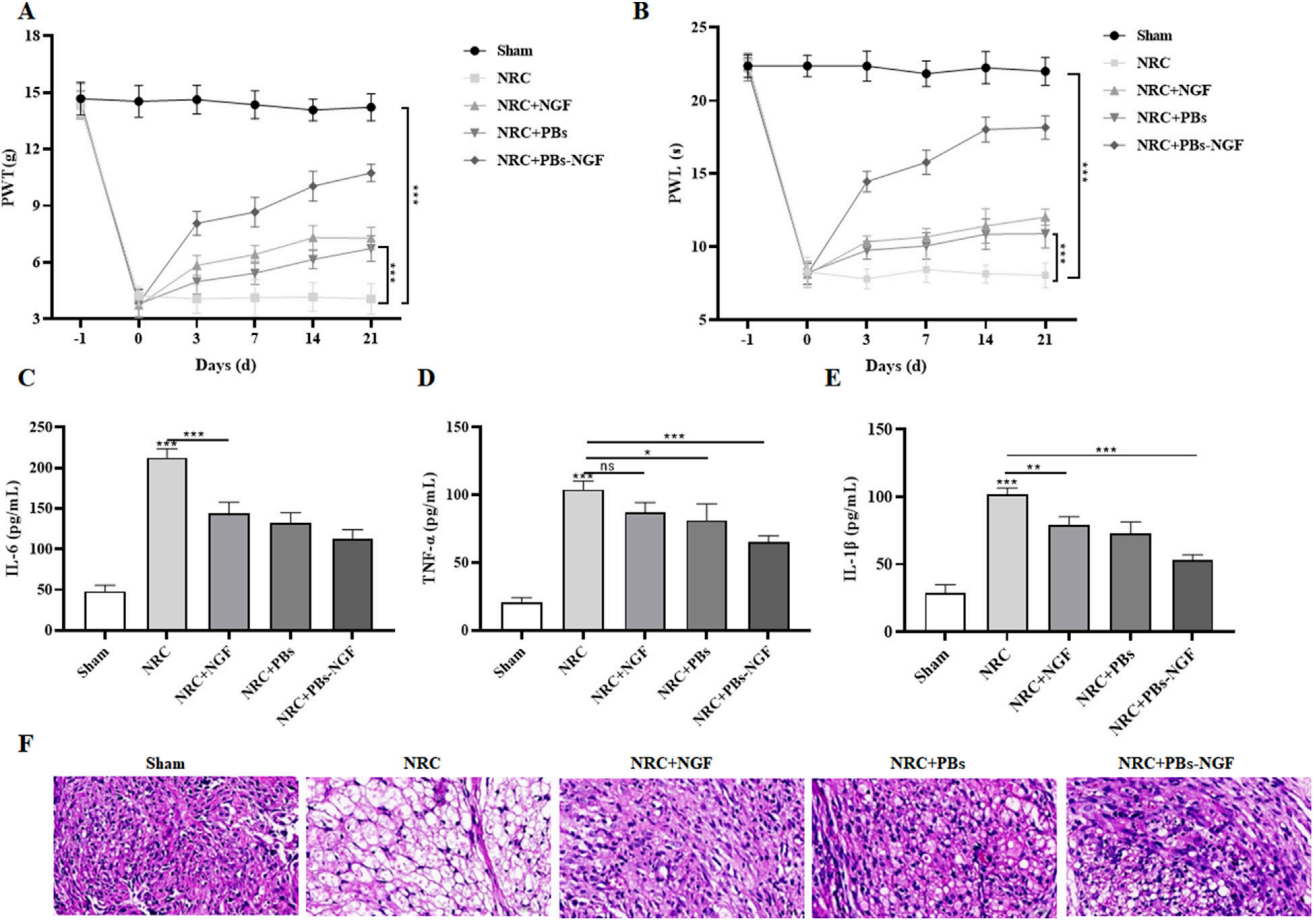
Dynamic change of paw withdrawal threshold (PWT) **(A)** and paw withdrawal latency (PWL) **(B)** in a nerve root compression (NRC) model of SD rats after free NGF, PBs nanoparticle, or PBs-NGF nanoparticle treatment **(A, B)**. n = 7 of each group. Concentrations of IL-6 **(C)**, TNF-α **(D)**, and IL-1β **(E)** in the serum of NRC model SD rats after free NGF, PBs nanoparticle, or PBs-NGF nanoparticle treatment **(C–E)**. H&E staining was used to observe the inflammatory infiltration of nerve roots in NRC model SD rats after free NGF, PBs nanoparticle, or PBs-NGF nanoparticle treatment **(F)**. *p < 0.05; **p < 0.01; ***p < 0.001. ns indicates no statistical difference.

To verify the modulating effects of PBs-NGF on NRC-induced pain behavior, we assessed behavioral motor performance of NRC severity by examining paw withdrawal threshold (PWT) and paw withdrawal latency (PWL) after treatment with PBs-NGF. Rats were injected intravenously with free NGF, free PBs, or PBs-NGF 2–4 days after NRC. The PWT and PWL tests were performed before NRC modeling and on days 0, 3, 7, 14, and 21 after NRC. The results in Figures 5A,B show that PWT and PWL were significantly reduced in rats after NRC compared with the results before NRC modeling, confirming successful NRC modeling. Then, in the PBs-NGF nanoparticle group, PWT and PWL were significantly increased after treatment, showing that PBs-NGF led to the NRC rats experiencing significant functional recovery.

Previous studies showed that NGF has anti-inflammatory and antioxidant effects and could reduce inflammatory reactions after nerve injury (Sencar et al., 2020). Based on this, we examined the inflammatory factors, including IL-6, TNF-α, and IL-1β, in the serum of NRC rats in each group. These inflammatory factors play an important role in NRC. The levels of IL-6, TNF-α, and IL-1β in the NRC group were much higher than those in the control group, indicating the severity of the disease. Fortunately, these upregulated inflammatory factors were significantly reduced with the help of PBs-NGF (Figures 5C–E). Subsequently, the inflammatory infiltration of nerve roots in NRC rats treated with PBs-NGF was observed by H&E staining. The nuclei of neurons in the normal group showed dense blue staining, while the nuclei of motor neurons in the model group showed light blue staining. Compared with the normal group, the number of motor neurons in the model group was significantly reduced, while the number of neurons in the PBs-NGF group was significantly increased (Figure 5F).

### 3.5 Biosafety of PBs and PBs-NGF

**FIGURE 6:**
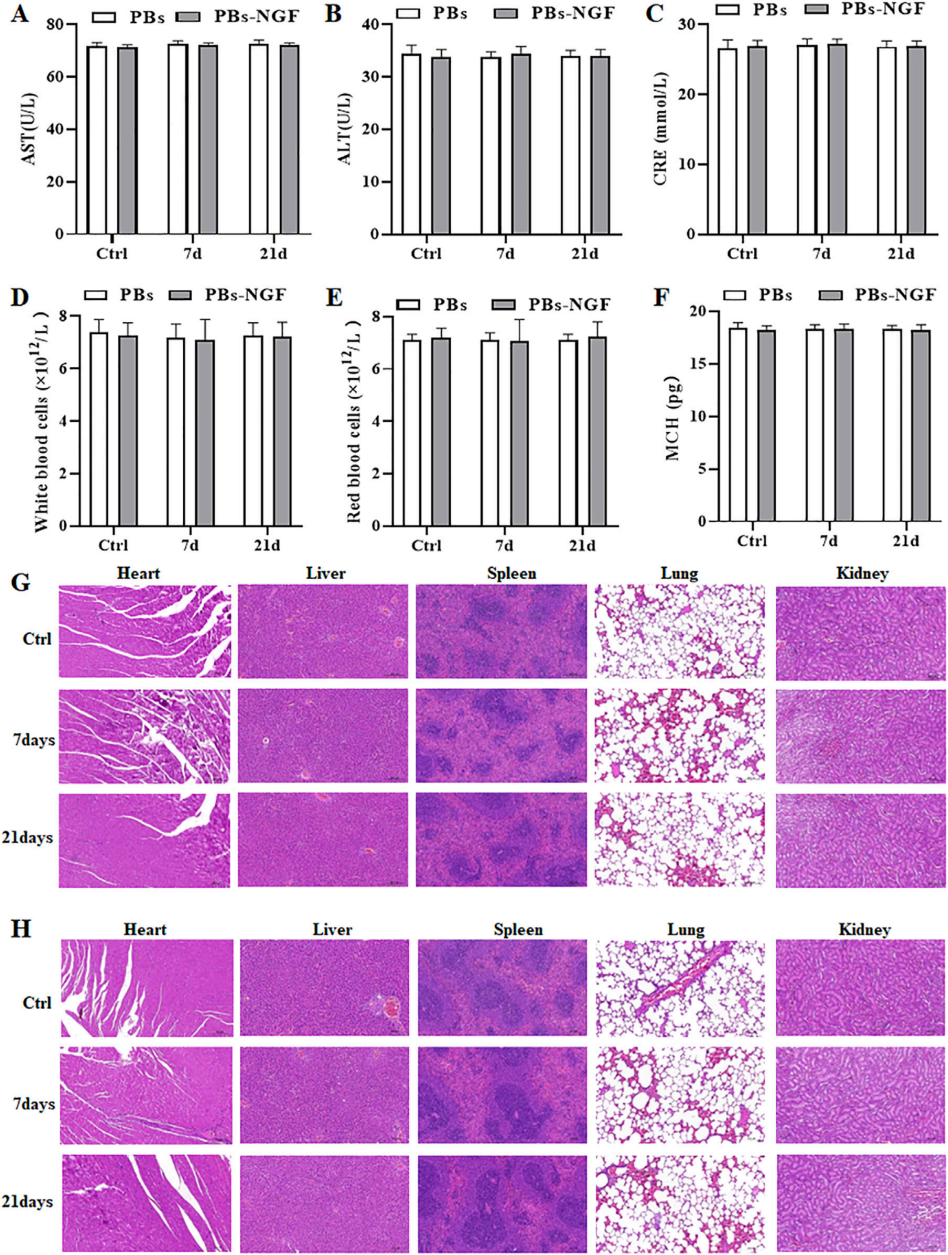
Serum biochemistry studies, including factors like AST **(A)**, ALT **(B)**, and CRE **(C)**. Routine blood studies, including factors like WBC **(D)**, RBC **(E)**, and MCH **(F)** of healthy rats 7 days or 21 days post-administration of PBs or PBs-NGF. Images of H&E-stained main organ tissues (including heart, liver, spleen, lungs, and kidney) resected from mice after 7 days and 21 days of treatment with PBs **(G)** or PBs-NGF **(H)**.

Further study of the biological safety of the PBs and PBs-NGF used a daily dose of 20 mg/kg for 21 days. Physiological saline was used for the control group. During these 21 days of treatment, all the experimental rats behaved normally and did not exhibit abnormal behaviors such as diarrhea, vomiting, and convulsions. The rats were terminally anesthetized after 21 days, and the main organs (liver, spleen, kidney, stomach, and colon) and blood were resected. Clinical and biochemical analysis revealed that ALT, AST, and CRE were maintained at the normal level (Figures 6A–C). No liver or kidney damage was noted during the experiment. In addition, WBC, RBC, MCH, and routine blood index levels were within the normal range for rats in the PBs and PBs-NGF nanoparticle treatment groups and the control group (Figures 6D–F). Central, liver, spleen, lung, and kidney tissues underwent H&E staining, and no samples showed obvious abnormalities (Figures 6G,H).

## 4 Conclusion

In this research, biocompatible PBs with multi-enzyme activity were successfully constructed and are effective reactive oxygen species scavengers that can alleviate harmful reactive oxygen species, including ·OH, H_2_O_2_, and ·OOH in H_2_O and O_2_ to relieve oxidative stress. Successfully coupled with NGF, the constructed PBs-NGF could promote PC12 cell proliferation and neural differentiation. In NRC rats, PBs-NGF can aggregate at the compressed nerve root and release NGF in high concentration for a long time through the inflammatory tissue targeted aggregation effect. In addition, PBs-NGF can effectively relieve pain caused by NRC and reduce inflammatory infiltration of nerve roots. This study provides a theoretical basis and practical guidance for long-term NGF treatment of neurological injury-related diseases. It offers a new treatment approach for clinicians to deal with various cases of nerve root function impairment.

In the future, we will improve and study the following aspects: First, we will supplement the experiments that are missing in the article, including the characterization of nanoparticles with an energy-dispersive spectrometer and the study of validation of the biocompatibility (containing the environmental safety data and other model systems). Second, we will optimize the nanomaterial drug delivery system, including modification, enhancement of drug loading rate, and whether it can load other drugs. Finally, we will further explore the molecular mechanism of PBs-NGF in relieving neuropathic pain.

## Data Availability

The original contributions presented in the study are included in the article/Supplementary Material; further inquiries can be directed to the corresponding authors.
